# Towards By-Product Utilisation of Pea Hulls: Isolation and Quantification of Galacturonic Acid

**DOI:** 10.3390/foods7120203

**Published:** 2018-12-10

**Authors:** Friederike Gutöhrlein, Stephan Drusch, Sebastian Schalow

**Affiliations:** Department of Food Technology and Food Material Science, Technische Universität Berlin, Königin-Luise-Strasse 22, 14195 Berlin, Germany; friederike.gutoehrlein@tu-berlin.de (F.G.); stephan.drusch@tu-berlin.de (S.D.)

**Keywords:** pea hulls, galacturonic acid, Saeman hydrolysis, TFA, EDTA, cellulose, polygalacturonase

## Abstract

In order to evaluate by-products from food processing as alternative raw materials for pectin extraction, their amount of galacturonic acid (GalA) has to be analysed as a marker for pectin content. In the present study, significant differences in GalA release using different digestion methods are shown for pea hulls, as an example of by-products with a high content of cellulose. Complete digestion of the fibre matrix was assumed for Saeman hydrolysis as a reference protocol. Significantly lower GalA release was achieved by a treatment with trifluoracetic acid (TFA). An alternative treatment with ethylenediaminetetraacetic acid (EDTA) at pH 11 followed by an enzymatic digestion at pH 4.5 using a combination of polygalacturonase (Vegazyme M) and cellulase (Celluclast 1.5L) resulted in a similar release of GalA compared to Seaman hydolysis. Pea hull samples, analysed by this alternative protocol, showed on average a GalA content of 11.2%. Therefore, pea hulls may serve as new raw material for pectin extraction.

## 1. Introduction

*Pisum sativum* belongs to the legume plant family (Leguminosae) and is used in various food and feed applications due to its high content of protein, starch and dietary fibre [1]. In 2014, global production of dried peas amounted to around 11 million tonnes. Canada, the Russian Federation and China were the main producers [2]. Nowadays, dried peas are processed in order to isolate pea starch or pea protein from those. Hulls, which represent about 10 to 14% of total pea mass [3,4], are the first by-product. Their commercial valorisation has been limited up to now.

In addition to their high content of cellulose of approximately 65% [5], pea hulls contain 9 to 15% of uronic acids [5,6,7]. Weightman et al. already showed that these uronic acids almost exclusively consist of galacturonic acid (GalA; 97%) [6]. Given that pectins mainly consist of GalA [8], GalA may be considered as a marker in order to identify alternative raw materials for commercial pectin extraction.

The quantification of GalA from plant materials by means of spectrophotometric or chromatographic methods requires a complete decomposition of the GalA containing polysaccharide prior to analysis. In case of isolated pectins this degradation step may be easily integrated into the analysis protocol as it is conducted within the method of Blumenkrantz and Asboe-Hansen [9]. Dissolved pectin is degraded in boiling concentrated sulfuric acid-tetraborate and the GalA units released from the polysaccharide react in the presence of *m*-hydroxydiphenyl to form a chromogen that is detected at 520 nm. In contrast, complex cell wall matrices often require extensive decomposition protocols in order to release GalA from the polymer network completely [10]. Therefore, different decomposition protocols for complex cell wall materials may result in divergent amounts of GalA [11,12,13].

Hydrolysis based on concentrated sulfuric acid (H_2_SO_4_) is generally known as Saeman hydrolysis and is applied for the complete digestion of cell wall matrices, including cellulose and hemicelluloses [14]. However, depending on temperature and molarity of the acid during digestion there is a degradation of pentoses by H_2_SO_4_, while GalA resists the hydrolysis under typically applied conditions within the Saeman protocol [12]. Therefore, Saeman hydrolysis has already been used as a reference method for the comparison of digestion methods for the determination of GalA [15]. Nevertheless, this method is inadequate for simultaneous determination of neutral sugars in polysaccharides and complex cell wall materials.

Another hydrolysis based on trifluoracetic acid (TFA) is commonly used for soluble and non-cellulosic materials. Furthermore, there is also a digestion of cellulose by TFA, especially at long reaction times [14]. No further degradation of GalA or neutral sugar was observed within a hydrolysis of six hours in 2M TFA at 100°C [12].

As an improvement of TFA hydrolysis, a subsequent treatment with enzymes and acid, which increases the release of GalA, has already been described. The yield of GalA was increased for pectin [12,16] as well as for cell wall materials from apples [17,18], but it strongly depended on type of enzyme used for the treatment. Another method for the release of GalA, which is based on an extraction with EDTA, was applied for cell wall materials from apples by Vetter [19]. EDTA solubilises pectin by complexation of divalent cations [20], which leads to an opening of the cell wall matrix and enhances enzymatic degradation at accessible sequences of pectin [21]. However, an evaluation in comparison to reference protocols like Saeman hydrolysis was not in the focus in the study of Vetter [19].

The present study aims at identifying an alternative method to Saeman hydrolysis for the release of GalA from pea hulls, which allows a further quantification of neutral sugars. Furthermore, the support of GalA release by commercial enzyme preparations during the digestion of fibre was evaluated. In this work, pea hulls are considered an example of complex cell wall materials with a high content of cellulose. In order to evaluate the potential of pea hulls as an alternative source for pectin extraction, pea varieties and different commercial samples of pea hull fibre were analysed regarding their GalA content.

## 2. Materials and Methods 

### 2.1. Material

All chemicals were of analytical grade and supplied by Carl Roth GmbH & Co. KG (Karlsruhe, Germany) and VWR International GmbH (Dresden, Germany). Enzyme preparations (Table 1) have been generously provided by Novozymes Switzerland AG (Dittingen, Switzerland) and Erbslöh Geisenheim AG (Geisenheim, Germany).

Commercial pea hull products have been supplied by Emsland Stärke GmbH (Emlichheim, Germany), Herbafood Ingredients GmbH (Werder, Germany), AM Nutrition AS (Stavanger, Norway) and Cosucra (Warcoing, Belgium). IGV GmbH (Nuthethal, Germany) and the Institute of Food Chemistry, University Hamburg (Hamburg, Germany) provided hulls of six pea varieties (Salamanca, Rocket, Starter, Navarro, James, Gregor).

### 2.2. Methods

At first, three chemical digestion methods were evaluated regarding GalA release: Saeman hydrolysis, TFA hydrolysis with and without additional enzyme treatment as well as EDTA hydrolysis with additional enzyme treatment. For these experiments, different polygalacturonases and combinations with cellulase were applied (Table 1). Experiments were executed with one batch of the commercial pea hull preparation PH 1000 (Emsland Stärke GmbH). After digestion, solid particles were separated by filtration. The permeate was diluted (1:10) before analysing GalA photometrically by the *m*-hydroxydiphenyl method [9] as follows: 0.5 mL of diluted sample was mixed with 3.0 mL of a 0.0125 M solution of sodium tetraborate in concentrated H_2_SO_4_ and heated in boiling water for 10 min. After cooling to room temperature, 50 µL of a 0.15% *m*-hydroxydiphenyl solution (in 0.5% NaOH) was added. The extinction of the solution was measured at a wavelength λ = 520 nm. A calibration curve was prepared by using galacturonic acid in various concentrations ranging from 2 to 40 µg/mL. All measurements were carried out in triplicate. All enzymatic digestions were executed with a solution of 2% (*v*/*v*) commercial enzyme preparation (Table 1). Furthermore, combinations of polygalacturonase and cellulase (P1 + C and P2 + C) in a ratio of 3:1 were prepared and applied. All digestions were executed in duplicate.

Saeman hydrolysis was conducted according to the procedure described by Pettolino et al. as a reference analysis for complete digestion [14]. Therefore, no further enzymatic digestion was used.

GalA release by TFA was conducted according to Pettolino et al. [14] with the following modification. Samples (50 mg) were digested in 2M TFA for one hour at 120 °C. For additional enzymatic treatment, the sample was suspended in 8 mL of acetic acid acetate buffer (pH 4.5) and hydrolysed by 0.01 mL enzyme solution for 48 h at 40 °C prior to TFA hydrolysis.

GalA release with EDTA was performed according to Vetter as a combination of EDTA extraction and enzymatic digestion [19]. Therefore, 100 mg of pea hull was suspended in 45 mL Na_2_-EDTA solution (0.5%). In case of a one-stage treatment, pH of the suspension was adjusted to 4.5 with acetic acid and 2 mL of enzyme solution was added. The sample was incubated for 15 h at room temperature and filtrated for further analysis. For a two-stage treatment, suspension of the sample in Na_2_-EDTA was additionally adjusted to pH 11.8 with sodium hydroxide (NaOH) solution for the complexation of cations and incubated for one hour at room temperature. Afterwards, pH was decreased to 4.5 and the sample was prepared as described for the one-stage treatment before.

As a second step, the hulls of six pea varieties as well as a further 13 commercial pea hull fibre products were analysed to get an overview of the GalA content of pea hulls. For this analysis, the best-performing alternative method of digestion, developed in the first part of this work, was applied.

For data analysis, a general factorial design was created using the factors “digestion method” with three levels (TFA, one-stage EDTA and two-stage EDTA) and “type of enzyme” with six levels (C, P1, P2, PLC and the combinations P1 + C, P2 + C). Analysis of variance (ANOVA) was conducted by Design Expert (Version 8.0.7.1) in order to identify significant main and interaction effects by comparison of least significant differences (LSD).

## 3. Results and Discussion

### 3.1. Comparison of Galacturonic Acid (GalA) Release

**Figure 1:**
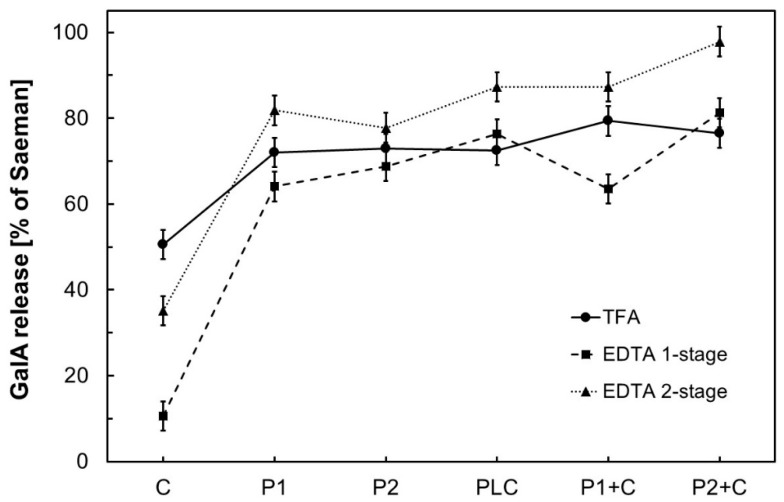
Release of galacturonic acid (GalA) in dependence of digestion method and applied enzyme (Table 1) in relation to Saeman hydrolysis (reference 100%). Results are based on analysis of variance (ANOVA) (mean ± least significant difference (LSD)).

Saeman hydrolysis resulted in highest release of GalA. Since complete hydrolysis of the cell wall matrix was assumed [14], the amount of detected GalA was set to 100% as reference. GalA release by the other methods was related to this value (Figure 1). It has to be mentioned that the method used for GalA quantification in pea fibre is based on the reaction of *m*-hydroxydiphenyl with uronic acids in general. Thus, apart from GalA, other uronic acids may be detected simultaneously. This would possibly lead to an overestimation of GalA in the respective sample. However, we regard this effect as marginal, since uronic acids in pea hulls mainly consist of GalA (>97%) as shown by Weightman et al. [6]. In addition, Blumenkrantz et al. reported that other cell wall neutral sugars do not interfere with uronic acid detection within this method [9].

The amount of GalA in pea hull fibre is significantly (*p* < 0.001) affected by the digestion method and by the type of enzyme used within the protocol (Table 2). Moreover, a significant interaction (*p* < 0.001) reveals, that the effect of the applied enzyme depends on the used digestion method. For instance, the digestion method had a high effect on GalA release if cellulase was applied alone. On the other hand, this effect was low in case of a single pectinase (P1, P2) treatment (Figure 1). Medium effects were found for the combined cellulase/pectinase treatments (P1 + C, P2 + C). Aiming at a high release of GalA by a suitable combination of enzyme and digestion method the main findings are discussed in the following section. 

If TFA-hydrolysis was applied without additional enzyme treatment, only 55.1% of GalA was released, compared to Saeman hydrolysis (result not shown). This incomplete release of GalA by 2 M TFA after 1 h has also been described by Emaga et al. for flaxseed mucilage [12]. While the addition of cellulase did not increase GalA release by TFA, a treatment with polygalacturonase significantly increased GalA release (Figure 1). Hence, an application of polygalacturonase is necessary for the release of GalA from pea fibre by TFA; cellulases can only support this reaction. The highest release of GalA by TFA hydrolysis was achieved by a subsequent treatment with polygalacturonase P1 and cellulase C (79.4% compared to the reference). Nevertheless, all experiments based on TFA digestion led to lower GalA contents than Saeman hydrolysis and should not be used as alternative protocol for GalA release from pea hulls, especially under considered conditions.

By EDTA treatment, GalA release by cellulase was significantly lower in comparison to a digestion with polygalacturonase (Figure 1). This effect was identified for a one-stage as well for a two-stage treatment. Consequently, an application of polygalacturonase is mandatory for extensive release of GalA from pea hull fibres within the EDTA protocol. Furthermore, GalA release by a two-stage treatment was significantly higher than by a one-stage treatment, independent of the enzyme used (Figure 1). An additional complexation step of cations at alkaline conditions as a part of the two-stage protocol significantly increased GalA release compared to a one-stage treatment. This result is in agreement with general investigations on EDTA, that showed better complexation of divalent cations at alkaline conditions [22,23]. This effect may be explained by a higher degree of dissociation of EDTA at increased pH. Furthermore, demethoxylation of polygalacturonates occurs under alkaline conditions, as it has already been described for pectins [24]. This demethoxylation enhances enzymatic depolymerisation of pectin via polygalacturonase [25,26], which may also result in an increased decomposition of pectic substances in complex cell wall materials. In the present study, highest GalA release using an alternative digestion protocol was realised by an EDTA treatment with a subsequent enzymatic digestion as combination of P2+C (98.9% of Saeman). Therefore, this protocol is comparable to Saeman hydrolysis for determination of GalA in pea hulls. As a consequence, this protocol was used to determine GalA contents in hulls of different pea varieties and commercial pea hull products.

### 3.2. GalA Content of Different Pea Hull Varieties

Pea hull samples of six different varieties showed a content of GalA between 10.9% and 11.7% (Table 3), which corresponds to an average of GalA of 11.2% in pea hulls. This range is smaller than described by Ali-Khan, who showed the variance of pea hull content and its composition in dependence of variety, cultivation region and year of harvest [3].

In the present study, pea hulls originated in the same year and from the same region, which may result in homogeneity between varieties. Commercial samples of pea hulls showed a GalA content between 9.7% and 12.4% (Table 3), which is within the natural range of pea composition, also shown for protein and starch content [27]. Moreover, the production process of pea hull fibre might influence the purity and, therefore, the content of GalA in pea hulls, which may explain the lower values of some commercial products.

In the present study, the GalA content of pea hull fibre is generally lower than that values of around 16% reported previously [6,28,29]. However, in these studies, an alcohol-insoluble residue was analysed, which may result in a higher content of GalA due to a washing out of low molecular polysaccharides and proteins.

## 4. Conclusions

In the present study, an alternative protocol was developed and compared to the commonly used Saeman hydrolysis as the reference for GalA release from plant cell wall materials. In this regard, a two-stage protocol resulted in the highest release of GalA with complexation of cations by EDTA at alkaline conditions and a subsequent enzymatic digestion at pH 4.5. The enzymatic treatment by polygalacturonase (Vegazyme M) and cellulase (Celluclast 1.5 L) offered a similar release of GalA from pea hull fibres compared to the reference method. In contrast to Saeman hydrolysis, a decomposition of pentoses is not expected for EDTA treatment in combination with the enzymes used. This might be favorable with regard to a further quantitative analysis of neutral sugars within the cell wall matrix. A study on hulls from different pea varieties showed a GalA content of 11.2% on average. Commercial pea hull fibre products varied in a larger range, which may be related to differences in environmental conditions during cultivation or different methods of processing.

For by-product utilisation of pea hulls as an alternative raw material for pectin extraction, further research on required conditions as well as on pectin composition and functionality should be realised.

## Figures and Tables

**Table 1:** Commercial enzyme preparations (manufacturers in brackets) and its declared activities.

Activity	P1 Pectinex Ultra SPL (Novozymes)	P2 Vegazym M (Erbslöh)	PLC Ultrazym AFP-L (Novozymes)	C Celluclast 1.5L (Novozymes)
Polygalacturonase 3.2.1.15	X	X	X	
Pectin lyase 4.2.2.10			X	
Cellulase 3.2.1.4			X	X

**Table 2:** ANOVA of effects of digestion method and applied enzyme (* *p*-values less than 0.05 indicate that model terms are significant).

Source	Sum of Squares	df	Mean Square	F-Value	*p*-Value
Model	14,215.75	17	836.22	103.32	<0.0001 *
A (digestion method)	1757.36	2	878.68	108.57	<0.0001 *
B (type of enzyme)	10,878.32	5	2175.66	268.82	<0.0001 *
AB	1580.07	10	158.01	19.52	<0.0001 *
Pure error	145.68	18	8.09		
Corr. total	14,361.43	35			

**Table 3:** GalA content of pea hulls (mean ± standard deviation (SD)); varieties in single-origin form and commercially available pea hull samples.

	GalA Content (%)
Salamanca	11.1 ± 0.1
Rocket	11.0 ± 0.3
Starter	10.9 ± 0.2
Navarro	11.0 ± 0.3
James	11.5 ± 0.2
Gregor	11.7 ± 0.2
Commercial samples (*n* = 14)	9.7 ± 0.1–12.4 ± 0.0
